# Kawasaki Disease Presenting as Acute Acalculous Cholecystitis

**DOI:** 10.5811/cpcem.2019.8.44255

**Published:** 2019-10-14

**Authors:** Demis N. Lipe, Lindsey C. Bridges

**Affiliations:** * MD Anderson Cancer Center, Department of Emergency Medicine, Houston, Texas; † Navicent Health, Department of Surgery, Macon, Georgia

## Abstract

Acute acalculous cholecystitis (AAC) is a rare, potentially serious disease that has been associated with Kawasaki disease (KD) in children. Studies suggest that patients presenting with severe abdominal symptoms secondary to KD have increased resistance to intravenous immunoglobulin (IVIG), and a higher rate of coronary artery aneurysms. We describe an eight-year-old boy who presented to the emergency department with severe abdominal pain and was diagnosed with AAC and KD. He was treated with IVIG and high-dose aspirin, achieving good response with complete symptom resolution. He had no coronary artery aneurysms or further complications and was discharged after three days.

## INTRODUCTION

Kawasaki disease (KD) is a pediatric vasculitis that typically presents with a set of non-specific symptoms, such as abdominal pain, vomiting, diarrhea, rash, cough, rhinorrhea, and irritability, which can obscure a correct diagnosis.^1^ Rarely, KD may present with an acute surgical abdomen caused by conditions such as acute acalculous cholecystitis (AAC), gallbladder hydrops, appendicular vasculitis, or hemorrhagic duodenitis.^2^ Patients who present with gastrointestinal symptoms as a manifestation of KD often have a delay in the diagnosis of KD and initiation of therapeutic interventions, and can be subjected to unnecessary surgical interventions.^3^ Further, KD is associated with coronary artery involvement in approximately 5% of affected patients.^4^

AAC is an inflammatory disease of the gallbladder that is associated with various systemic illness including KD.^5^ Although AAC is usually benign and self-limited, in rare instances it may require surgical intervention to avoid life-threatening complications such as sepsis and death.^5^,^6^ AAC has been reported to be a marker of more severe disease and is associated with resistance to intravenous immunoglobulin (IVIG, part of the standard treatment regimen for KD) and increased risk for coronary artery lesions.^3^,^4^ Thus, prompt recognition of AAC is imperative. When AAC is diagnosed in the emergency department (ED), emergency physicians should consider the possibility of an underlying systemic illness, especially in children, to avoid delay in diagnosis and treatment and to prevent coronary lesions.^3^

To our knowledge, no instances of KD have been reported in the emergency medicine literature. Here, we present a case report describing a pediatric patient with AAC who responded well to IVIG and had no associated coronary lesions.

## CASE REPORT

An eight-year-old boy was brought to the ED with right-sided abdominal pain, diarrhea, vomiting, and fever for the prior week. He had been evaluated in the ED on the first day of his illness and diagnosed with a viral infection. On day two of the illness the child had been evaluated by his pediatrician, who suspected a urinary tract infection on the basis of urinalysis results showing pyuria. On arrival at the ED, the patient had a blood pressure of 100/60 millimeters of mercury, a pulse rate of 96 beats per minute, a respiratory rate of 20 breaths per minute with oxygen saturation of 98% on room air, and an oral temperature of 98.2 degrees Fahrenheit (36.7 degrees Celsius). Physical examination revealed injected conjunctiva, desquamation of the lips, and tenderness of the right upper and lower abdominal quadrants, with rebound and guarding.

**Image 1:**
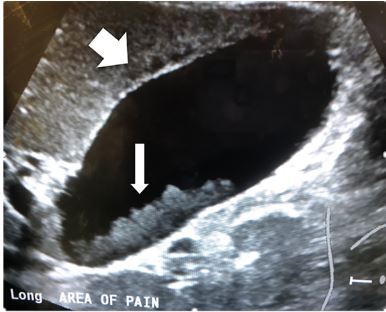
Formal ultrasonographic image showing a distended gallbladder in long axis with scant pericholecystic fluid (thick arrow) and sludge (thin arrow).

**Image 2:**
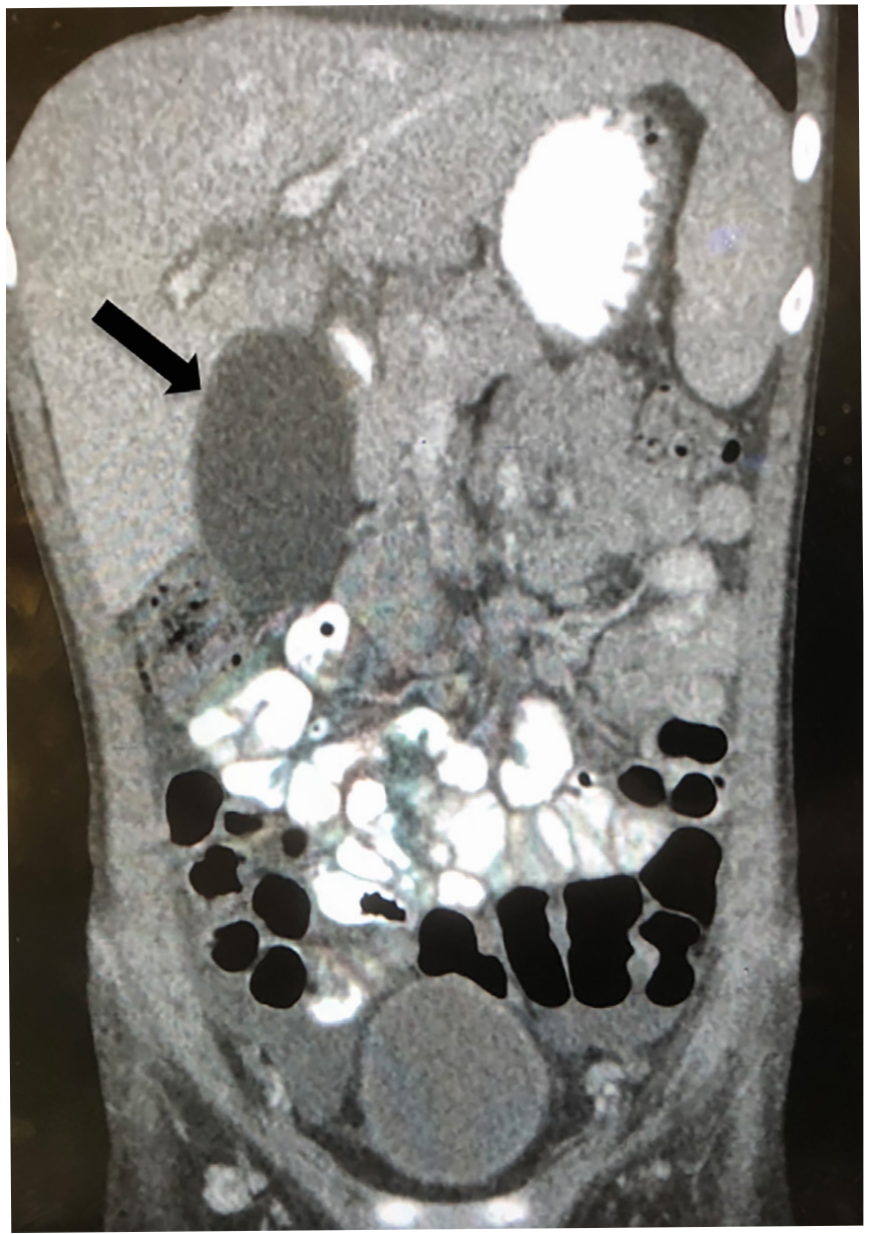
Computed Tomography showing distended gallbladder (arrow).

Laboratory studies showed a white blood cell count of 13,200 × 10^3^ per microliter (mcL) (4.5–14.5×10^3^/mcL), an erythrocyte sedimentation rate 28 millimeters per hour (1–13 mm/hr), a C-reactive protein level 6.2 milligrams per deciliter (mg/dL) (3–5 mg/dL), a total bilirubin level of 3.6 mg/dL (0.2–1.2mg/dL) with direct bilirubin of 2.8 mg/dL (<0.03 mg/dL), an alkaline phosphatase level of 428 units per liter (U/L) (35–104 U/L), and gamma-glutamyl transferase levels of 102 U/L (9–48 U/L). Formal abdominal ultrasonography revealed a distended gallbladder with scant pericholecystic fluid and sludge (Image 1). Computed tomography showed a distended gallbladder (Image 2).

A diagnosis of KD with AAC was made, and the patient was started on oral high-dose aspirin. He was transferred to a tertiary care center where he also received IVIG. All symptoms improved with treatment. His echocardiogram did not show any evidence of coronary artery aneurysms. The patient was discharged home with no further complications after hospital day three.

## DISCUSSION

KD is a common cause of pediatric vasculitis.^7^ Nonetheless, distinguishing KD from other febrile illness remains one of the biggest challenges that emergency physicians face.^8^ In the United States KD has surpassed rheumatic fever as the leading cause of acquired heart disease in children, affecting 19 per 100,000 children under the age of five.^9^,^10^

The clinical criteria for the diagnosis of KD include at least five days of fever plus at least four of five principal clinical features: polymorphous rash; oral changes; bilateral conjunctival injection; cervical lymphadenopathy; and extremity changes.^1^ If four or five of the clinical criteria are met, physicians may proceed to treatment; however, if only two or three of the five principal clinical criteria are met but clinical suspicion remains high, supplemental laboratory findings may aid diagnosis. Typically, complete blood count, hepatic panel, C-reactive protein level, erythrocyte sedimentation rate, and urinalysis are sufficient to supplement the two or three principal clinical criteria to enable the diagnosis. Such cases are much less common and are often referred to as “incomplete” or “atypical” KD.^1^,^7^

**CPC-EM Capsule**

What do we already know about this clinical entity?

*Patients presenting with severe abdominal pain secondary to Kawasaki disease (KD) have increased resistance to intravenous immunoglobulin and a higher rate of coronary aneurysms*.

What makes this presentation of disease reportable?

*The patient presented with acute acalculous cholecystitis (AAC)secondary to KD and had no coronary artery aneurysms; he responded well to intravenous immunoglobulin*.

What is the major learning point?

*AAC in children is often associated with systemic illness, such as KD. Medical treatment rather than immediate surgical intervention is preferred*.

How might this improve emergency medicine practice?

*When AAC is diagnosed in the emergency department an underlying systemic illness such as KD should be sought to avoid delay in diagnosis and treatment*.

Cardiac complications such as coronary artery aneurysm are the main concern with KD; nonetheless, other organ systems can be affected.^11^,^12^ For example, gastrointestinal (GI) symptoms with hepatobiliary abnormalities are the initial presentation in some patients.^5^ Atypical signs and symptoms that should prompt clinical suspicion for KD are hepatic dysfunction, gallbladder hydrops, jaundice, cholestasis, paralytic ileus, and AAC.^2^,^7^ Yi et al. noted that gallbladder distention alone in patients with KD is associated with coronary artery complications.^6^ A 2018 multicenter study conducted in Italy showed that GI symptoms as a manifestation of KD indicate greater risk for severe coronary lesions.^3^ Other factors included delayed treatment, low albumin level, and age younger than six months.^3^

Although AAC is uncommon in pediatric patients, recognizing it early is vital given the high incidence of coronary artery aneurysm associated with it.^6^ For a diagnosis of AAC, two of four ultrasonic criteria must be met: gallbladder distention; increased wall thickness (>3.5 mms); presence of sludge; or presence of pericholecystic fluid.^4^ This diagnosis can be made by point-of-care ultrasound in the ED.

Some have suggested that medical treatment, rather than immediate surgical intervention, is the preferred way to manage AAC in children with KD.^6^,^13^ The standard medical treatment for KD is high-dose aspirin (80–100 mg/kg/day) in conjunction with four doses of IVIG.^1^ Although aspirin is thought to have antiplatelet and anti-inflammatory effects, it does not reduce the risk for coronary artery aneurysm formation. In contrast, IVIG has been shown to reduce this risk, although it is most effective when administered within 7–10 days of illness onset.^1^ Even so, a retrospective study by Chen et al.^4^ found that KD patients with AAC treated with IVIG were more likely to be IVIG-resistant than were KD patients without AAC, thus bringing into question whether patients with AAC should receive IVIG therapy.

## CONCLUSION

Because KD may present similarly to other benign or potentially deadly diseases, it remains a challenging disease for emergency physicians to recognize. It is imperative that they be aware of the unusual presentations of the disease, such as AAC, and its association with coronary aneurysms. KD must always be considered in the differential diagnosis of a child with prolonged fever. The treatment of AAC in a child with KD is initially medical rather than surgical.

## References

[1] McCrindle BW, Rowley AH, Newburger JW (2017). Diagnosis, Treatment, and Long-Term Management of Kawasaki Disease: A Scientific Statement for Health Professionals From the American Heart Association. Circulation.

[2] Zulian F, Falcini F, Zancan L (2003). Acute surgical abdomen as presenting manifestation of Kawasaki disease. J Pediatr.

[3] Fabi M, Corinaldesi E, Pierantoni L (2018). Gastrointestinal presentation of Kawasaki disease: a red flag for severe disease?. Plos One.

[4] Chen CJ, Huang FC, Tiao MM (2012). Sonographic gallbladder abnormality is associated with intravenous immunoglobulin resistance in Kawasaki disease. Sci World J.

[5] Yi DY, Chang EJ, Kim JY (2016). Age, predisposing diseases, and ultrasonographic findings in determining clinical outcome of acute acalculous inflammatory gallbladder diseases in children. J Korean Med Sci.

[6] Yi DY, Kim JY, Choi EY (2014). Hepatobiliary risk factors for clinical outcome of Kawasaki disease in children. BMC Pediatr.

[7] Newburger JW, Takahashi M, Gerber MA (2004). Diagnosis, treatment, and long-term management of Kawasaki disease: a statement for health professionals from the Committee on Rheumatic Fever, Endocarditis and Kawasaki Disease, Council on Cardiovascular Disease in the Young, American Heart Association. Circulation.

[8] Stembergeng ML, Papic N, Sestan M (2018). Challenges in early diagnosis of Kawasaki disease in the pediatric emergency department: differentiation from adenoviral and invasive pneumococcal disease. Wien Klin Wochnschr.

[9] Taubert Ka, Rowley AH, Shulman ST (1991). Nationwide survey of Kawasaki disease and acute rheumatic fever. J Pediatr.

[10] Saguil A, Fargo M, Grogan S (2015). Diagnosis and management of Kawasaki disease. Am Fam Physician.

[11] Kato H, Sugimara T, Akagi T (1996). Long-term consequences of Kawasaki disease. A 10-to 21-year follow-up study of 594 patients. Circulation.

[12] Dajani AS, Taubert KA, Gerber MA (1993). Diagnosis and therapy of Kawasaki disease in children. Circulation.

[13] Hou JW, Chang MH, Wu MH (1989). Kawasaki disease complicated by gallbladder hydrops mimicking acute abdomen: a report of three cases. Zhonghua Min Guo Xiao Er Ke Yi Xue Hui Za Zhi.

